# Co-occurring health behaviors and mental health outcomes among a large, aging US population

**DOI:** 10.3389/fpubh.2026.1817690

**Published:** 2026-05-12

**Authors:** Kathryn E. Chiang, Erika Rees-Punia, Sicha Chantaprasopsuk, Alpa V. Patel, Lauren C. Bates-Fraser, Marissa M. Shams-White, Tamora A. Callands, Jessica L. Muilenburg, Heather M. Padilla

**Affiliations:** 1 Department of Population Science, American Cancer Society, Atlanta, GA, United States; 2 Department of Health Promotion and Behavior, College of Public Health, University of Georgia, Athens, GA, United States; 3 Winship Cancer Institute, Emory University, Atlanta, GA, United States

**Keywords:** alcohol, anxiety, body mass index, Cancer Prevention Study-3, depression, diet, physical activity, ACS Guideline Score

## Abstract

**Introduction:**

This study aimed to examine longitudinal associations of alignment to the American Cancer Society (ACS) Guidelines for Cancer Prevention in 2015 and self-reported anxiety and depression in 2021.

**Methods:**

Participants included 88,643 women (79%) and 23,373 men (21%) with a mean (SD) age of 53 (10) in the Cancer Prevention Study-3. The ACS Guideline Score captures alignment to guidelines for co-occurring health behaviors including body mass index, physical activity, diet quality, and alcohol consumption, on a range from 0-to-8; with higher scores indicating greater alignment. Self-reported anxiety and depression symptoms were measured via the Patient Health Questionnaire-4 (PHQ-4). Multivariable logistic regression models were used to calculate odds ratios and 95% confidence intervals. Further sensitivity analyses included: (1) excluding participants with prevalent depression and/or anxiety and those taking medications for depression and/or anxiety at baseline (*n* = 66,594); and (2) stratifying by level of change in PHQ-4 during the COVID-19 pandemic.

**Results:**

Among 112,016 participants, 32% reported both depression and anxiety symptoms, 10% reported anxiety symptoms, and 7% had symptoms of depression at follow-up. Participants with higher ACS Guideline Scores in 2015 were less likely to experience symptoms of depression and/or anxiety in 2021 compared to individuals with lower scores (OR = 0.60; 95% CI: 0.57–0.63). Analyses restricted to those without anxiety or depression in 2015 resulted in attenuated, though still statistically significant findings.

**Conclusion:**

These findings suggest lifestyles aligned with the ACS Guidelines for Cancer Prevention are associated with a lower likelihood of future symptoms of depression and anxiety.

## Introduction

In 2022, one in five US adults (59.3 million) had a mental illness (1); leading to over $280 billion in annual healthcare costs for mental health services (2). Stemming from a multitude of social, psychological, and biological factors (1, 3–5), depression and anxiety are two of the most common mental health disorders (4, 5). Both depression and anxiety increase the risk for heart diseases (6) and suicide (7), disrupting daily functioning, quality of life (8), and longevity (9). Comprehensive observational evidence suggests that individual modifiable health behaviors such as diet (10, 11), physical activity (PA) (12, 13), alcohol consumption (14), and smoking (15) are key influential factors for depression and anxiety (16). Furthermore, clusters of unhealthy behaviors (i.e., smoking, frequent alcohol use, poor diet, and sedentary behaviors) demonstrate synergistic effects and are linked to increasing an individual’s risk of anxiety and depression (17, 18), yet most studies focus on singular behaviors (17).

There may be a bidirectional relationship between mental health and health behaviors (19, 20). For example, a cumulative exposure analysis demonstrated PA is associated with reduced anxiety and depression; conversely, exposure to depression and anxiety symptoms is associated with a decreased levels of PA (19). A small number of studies have focused on multiple health behaviors (i.e., not smoking, regular PA, maintaining a healthy body weight, and consuming a diet high in fruits and vegetables) and report a relationship with better mental health outcomes; however, these studies are cross-sectional (21). Few studies have evaluated the longitudinal relationship between multiple health behaviors and mental health; thus, it is unclear how the combination of multiple health behaviors impact future depression and anxiety outcomes.

The 2020 American Cancer Society (ACS) Guideline for Cancer Prevention provides evidence-based recommendations for reducing cancer risk related to promoting overall health and shares many features with other organizations’ guidelines (22–25). It includes recommendations for body mass index (BMI), PA, diet, and alcohol consumption. The ACS Guideline Score allows researchers to study alignment with the guideline and can serve as a measure of co-occurring health behaviors (26). Alignment to the ACS Guideline has been demonstrated among healthy adult populations to be associated with reduced all-cause, cancer, and cardiovascular disease mortality (27) and social determinants of health (28); nonetheless, its effects on depression and anxiety have not been investigated.

The present study aims to examine longitudinal associations between co-occurring health behaviors captured by ACS Guideline Scores in 2015 and self-reported anxiety and depression in 2021 in a large, nationwide prospective cohort of aging US adults. A secondary exploratory aim examined associations between individual sub-score components and depression/anxiety symptoms to better understand which may be driving associations in the primary analysis.

## Materials and methods

### Study population and design

The Cancer Prevention Study-3 (CPS-3) is an ongoing prospective cohort study on cancer incidence and mortality (29). Nearly 304,000 participants aged 30 to 65 years with no cancer history (except basal or squamous cell skin cancer) were enrolled at ACS fundraising and recruitment events between 2006 and 2013. CPS-3 participants completed baseline surveys on demographics, lifestyle, and health history; and receive repeat surveys every 3 years to update exposure information. Further details regarding participant characteristics, cohort descriptions, and recruitment are described elsewhere (29). The Institutional Review Board at Emory University approved all aspects of CPS-3. All participants provided written informed consent. This study followed the Reporting of Observational Studies in Epidemiology (STROBE) reporting guideline.

A total of 186,638 participants returned the 2015 follow-up survey. Individuals were excluded if they were missing information on race/ethnicity (*n* = 1,124); underweight or missing BMI (*n* = 2,968), physical activity (*n* = 12,513), alcohol use (*n* = 145), and missing/incomplete (30) Food Frequency Questionnaire (*n* = 17,111) data from 2015; for unreturned CPS-3 follow-up surveys in 2021 (*n* = 34,383) or incomplete 2021 Patient Health Questionnaire-4 (*n* = 2,420); and for pregnancy at the time of baseline and/or 2015 survey collection (*n* = 3,958).

### Measures

#### ACS Guideline Score

Detailed methodology of the ACS Guideline Score were previously described (26, 28). Briefly, each guideline component was weighed equally and scored from 0-to-2; with 2, 1, and 0 indicating meeting/exceeding, partial, and zero alignment to the guideline, respectively. Each sub-score was then summed to create a total ACS Guideline Score ranging from 0-to-8, with higher scores indicating better alignment and health behaviors.

BMI was calculated at two timepoints to assess weight maintenance, using height and weight from the 2006–2013 enrollment survey and weight from 2015 follow-up survey, leveraging CPS-3 data availability to provide a longer-term measure of BMI in adulthood (26). The highest score of 2 was given to individuals with a normal BMI of 18.5 to < 25 kg/m^2^ at both timepoints; 0 was given to individuals with a BMI ≥ 30 kg/m^2^ (i.e., with obesity) at one or both timepoints; and a score of 1 was given to all other BMI combinations.

Using self-reported data collected in 2015, minutes of moderate-to-vigorous physical activity (MVPA) per week were converted to metabolic equivalent of task (MET)-hours/week; this included walking, biking, tennis, swimming, and other aerobic activities. Individuals exceeding PA guidelines (≥15 MET-h/wk) received a score of 2, meeting guidelines (7.5 to <15 MET-h/wk) received a 1, and inactive individuals (<7.5 MET-h/wk) received a score of 0.

Dietary intake was categorized into survey- and sex-specific quartiles for intake and variety of fruits and vegetables, whole grains, red/processed meats, sugar-sweetened beverages (SSBs), and highly processed or refined grains (HPF/RG) (26, 31). Participants in the highest quartiles for fruit and vegetable intake and variety, and whole grain intake received higher scores (0–3 points each), while those with the highest intake of red/processed meats, SSBs, and HPF/RG were inversely scored and received the lowest scores (3–0 points combined). Dietary sub-scores were then summed to yield a total diet score ranging from 0-to-12, with higher scores indicating better diet quality (26, 31). To align with the weighing of other guideline components, diet scores were rescaled based on tertile distributions in the study population to a 0-to-2 point scale.

Self-reported alcohol consumption was scored where individuals who consumed >1 drink/day for females and >2 drinks/day for males received a 0; drinking >0 – ≤ 1 drink/day for females and >0 – ≤ 2 drinks/day for males earned a 1 (i.e., moderate consumption (22)); and nondrinkers received a score of 2.

#### Depression and anxiety outcomes

The four-item Patient Health Questionnaire (PHQ-4) from the 2021 CPS-3 survey was used to measure self-reported symptoms of anxiety and depression (32). PHQ-4 consists of two items for depression (PHQ-2) and two items for Generalized Anxiety Disorder (GAD-2) symptoms (32). Previous studies support the reliability and validity of using PHQ-4 (Cronbach’s alpha = 0.85) and its subscales, PHQ-2 (Cronbach’s alpha = 0.81) and GAD-2 (Cronbach’s alpha = 0.82) as alternatives to their lengthier counterparts, PHQ-9 and GAD-7 (33–35). PHQ-4 items are detailed in Supplementary eTable 1. Total PHQ-4 scores ranged from 0-to-12 and were determined by adding together the scores of each of the four items, with higher scores indicating greater levels of depression and anxiety (32). PHQ-4 scores were categorized as normal (0–2; referent), mild (3–5), moderate (6–8), and severe (9–12), and were collapsed into normal (0–2) and mild-to-severe (3–12) because of sparsity. Sub-scores for PHQ-2 and GAD-2 were used to indicate high ( ≥ 3) or low (<3) depression or anxiety symptoms. The PHQ-4 assesses symptoms at screening-level thresholds and prevalence estimates reflect elevated symptom burden rather than clinical diagnoses of depression or anxiety.

#### Covariates

Numerous sociodemographic characteristics are known risk factors for health behaviors, anxiety, and depression; therefore, *a priori* covariates were selected (36–38). Demographic information for sex, age, and race/ethnicity were measured at baseline. Work status, annual income, education level, marital status, and energy intake were assessed using the 2015 CPS-3 survey.

#### Statistical analysis

Associations between ACS Guideline Scores (total and individual sub-scores) and self-reported mental health outcomes were evaluated using multivariate logistic regression. All models were adjusted for aforementioned covariates. Tail end distributions of ACS Guideline Scores (0, 1, and 2 for lowest scores; 7–8 for highest scores) were collapsed and the lowest score of 0–2 was used as the reference group. In secondary models of individual sub-score exposures, the referent was a score of 0; thus, all results describe the odds of a higher score over the lowest score.

A sensitivity analysis was performed to address concerns of reverse causality by excluding participants with prevalent self-reported depression and/or anxiety and participants taking medications for depression and/or anxiety in 2015 and/or 2021.

To account for the occurrence of the COVID-19 pandemic during the study follow-up years, PHQ-4 data pertaining to participants’ responses to the pandemic was assessed. In 2021, the PHQ-4 question was modified to, “*During the COVID-19 pandemic, how often have you been bothered by the following problems?”* with the following response options: ‘Less often,’ ‘About the same,’ and ‘More often’ than before the pandemic. Responses were summed on a range from −4 to 4, with more negative scores indicating PHQ-4 (i.e., depression and anxiety symptoms) decreased and more positive scores indicating PHQ-4 increased during COVID-19. A stratified analysis by changes in PHQ-4 scores during COVID-19 was considered, with strata as increased, decreased, and unchanged. All analyses were conducted in R Studio Pro 2024.04.1 running R version 4.4.0 with *p*-values <0.05 denoting statistical significance.

## Results

Sociodemographic and health characteristics of participants are detailed in Table 1. The final analytic sample included 112,016 participants; 79% (*n* = 88,643) were female with a mean (SD) age of 53 (10) years. By the 2021 survey, 6.6% of participants had symptoms of depression (*n* = 7,343), 10% experienced symptoms of anxiety (*n* = 11,404), and 32% reported both depression and anxiety symptoms (*n* = 36,147). Nearly 72% (*n* = 12,108) of participants with the highest ACS Guideline Scores of 7–8 in 2015 had normal PHQ-4 scores in 2021.

**Table 1:** Participant characteristics by Patient Health Questionnaire-4 (PHQ-4).

	Participants, No. (%)				
	Overall (*n* = 112,016)	PHQ-4 categories			
Characteristic		Normal (*n* = 75,869)	Mild (*n* = 28,574)	Moderate (*n* = 5,558)	Severe (*n* = 2,015)
Age, mean (SD)	53 (9.5)	53 (9.3)	51 (9.7)	49 (9.8)	49 (9.7)
Sex					
Female	88,643 (79.1%)	58,125 (76.6%)	23,958 (83.8%)	4,817 (86.7%)	1,743 (86.5%)
Male	23,373 (20.9%)	17,744 (23.4%)	4,616 (16.2%)	741 (13.3%)	272 (13.5%)
Race and ethnicity					
Asian, Native Hawaiian, Pacific Islander, or American Indian	2,741 (2.4%)	1,821 (2.4%)	702 (2.5%)	156 (2.8%)	62 (3.1%)
Black	2,207 (2.0%)	1,541 (2.0%)	502 (1.8%)	109 (2.0%)	55 (2.7%)
Latino (all races)	5,753 (5.1%)	3,774 (5.0%)	1,531 (5.4%)	325 (5.8%)	123 (6.1%)
White	100,863 (90.0%)	68,462 (90.2%)	25,709 (90.0%)	4,934 (88.8%)	1,758 (87.2%)
Other^a^	452 (0.4%)	271 (0.4%)	130 (0.5%)	34 (0.6%)	17 (0.8%)
Work status					
Full time	75,344 (67.3%)	50,853 (67.0%)	19,383 (67.8%)	3,774 (67.9%)	1,334 (66.2%)
Part time	13,626 (12.2%)	9,275 (12.2%)	3,469 (12.1%)	670 (12.1%)	212 (10.5%)
Retired	13,919 (12.4%)	10,242 (13.5%)	3,093 (10.8%)	442 (8.0%)	142 (7.0%)
Other	7,047 (6.3%)	4,186 (5.5%)	2,060 (7.2%)	529 (9.5%)	272 (13.5%)
Unknown/missing	2,080 (1.9%)	1,313 (1.7%)	569 (2.0%)	143 (2.6%)	55 (2.7%)
Income, $					
<50,000	15,709 (14.0%)	9,518 (12.5%)	4,482 (15.7%)	1,178 (21.2%)	531 (26.4%)
50,000 to <75,000	19,312 (17.2%)	12,529 (16.5%)	5,282 (18.5%)	1,092 (19.6%)	409 (20.3%)
75,000 to <100,000	19,732 (17.6%)	13,262 (17.5%)	5,169 (18.1%)	977 (17.6%)	324 (16.1%)
100,000 to <125,000	18,294 (16.3%)	12,486 (16.5%)	4,668 (16.3%)	841 (15.1%)	299 (14.8%)
125,000 or more	37,269 (33.3%)	26,801 (35.3%)	8,620 (30.2%)	1,419 (25.5%)	429 (21.3%)
Unknown/missing	1,700 (1.5%)	1,273 (1.7%)	353 (1.2%)	51 (0.9%)	23 (1.1%)
Education level					
High school or less	6,945 (6.2%)	4,680 (6.2%)	1,737 (6.1%)	379 (6.8%)	149 (7.4%)
Some college or 2-y degree	27,906 (24.9%)	18,434 (24.3%)	7,286 (25.5%)	1,525 (27.4%)	661 (32.8%)
College graduate	39,908 (35.6%)	27,064 (35.7%)	10,227 (35.8%)	1,955 (35.2%)	662 (32.9%)
Graduate degree	36,961 (33.0%)	25,473 (33.6%)	9,262 (32.4%)	1,686 (30.3%)	540 (26.8%)
Unknown/missing	296 (0.3%)	218 (0.3%)	62 (0.2%)	13 (0.2%)	3 (0.1%)
Marital status					
Married or living with partner	86,093 (76.9%)	59,540 (78.5%)	21,314 (74.6%)	3,939 (70.9%)	1,300 (64.5%)
Never married	8,364 (7.5%)	4,951 (6.5%)	2,568 (9.0%)	590 (10.6%)	255 (12.7%)
Divorced, separated, or widowed	16,258 (14.5%)	10,463 (13.8%)	4,377 (15.3%)	983 (17.7%)	435 (21.6%)
Unknown/missing	1,301 (1.2%)	915 (1.2%)	315 (1.1%)	46 (0.8%)	25 (1.2%)
ACS Guideline Scores					
0–2	14,127 (12.6%)	8,528 (11.2%)	4,164 (14.6%)	1,012 (18.2%)	423 (21.0%)
3	15,952 (14.2%)	10,168 (13.4%)	4,431 (15.5%)	964 (17.3%)	389 (19.3%)
4	21,267 (19.0%)	14,175 (18.7%)	5,586 (19.5%)	1,101 (19.8%)	405 (20.1%)
5	23,671 (21.1%)	16,462 (21.7%)	5,839 (20.4%)	1,021 (18.4%)	349 (17.3%)
6	20,227 (18.1%)	14,428 (19.0%)	4,705 (16.5%)	823 (14.8%)	271 (13.4%)
7–8	16,772 (15.0%)	12,108 (16.0%)	3,849 (13.5%)	637 (11.5%)	178 (8.8%)
BMI score^b^					
0 ( ≥ 30.0 kg/m^2^ at any time point)	33,138 (29.6%)	20,714 (27.3%)	9,440 (33.0%)	2,101 (37.8%)	883 (43.8%)
1 (Other combinations)	37,323 (33.3%)	25,959 (34.2%)	9,079 (31.8%)	1,670 (30.0%)	615 (30.5%)
2 (18.5–<25.0 kg/m^2^ at both time points)	41,555 (37.1%)	29,196 (38.5%)	10,055 (35.2%)	1,787 (32.2%)	517 (25.7%)
Physical activity score					
0 (<7.5 MET-h/wk)	34,209 (30.5%)	21,650 (28.5%)	9,545 (33.4%)	2,133 (38.4%)	881 (43.7%)
1 (7.5 - < 15.0 MET-h/wk)	11,268 (10.1%)	7,494 (9.9%)	3,000 (10.5%)	569 (10.2%)	205 (10.2%)
2 ( ≥ 15 MET-h/wk)	66,539 (59.4%)	46,725 (61.6%)	16,029 (56.1%)	2,856 (51.4%)	929 (46.1%)
Diet score^c^					
0 (first tertile)	34,401 (30.7%)	22,341 (29.4%)	9,217 (32.3%)	2,054 (37.0%)	789 (39.2%)
1 (second tertile)	36,550 (32.6%)	24,838 (32.7%)	9,352 (32.7%)	1,734 (31.2%)	626 (31.1%)
2 (third tertile)	41,065 (36.7%)	28,690 (37.8%)	10,005 (35.0%)	1,770 (31.8%)	600 (29.8%)
Alcohol score					
0 (>1 drink/d for women, >2 drinks/d for men)	8,763 (7.8%)	5,913 (7.8%)	2,263 (7.9%)	441 (7.9%)	146 (7.2%)
1 ( ≤ 1 drink/d for women, ≤ 2 drinks/d for men)	73,724 (65.8%)	50,342 (66.4%)	18,739 (65.6%)	3,486 (62.7%)	1,157 (57.4%)
2 (0 drink/d)	29,529 (26.4%)	19,614 (25.9%)	7,572 (26.5%)	1,631 (29.3%)	712 (35.3%)
Depression symptoms (PHQ-2) in 2021					
Low	104,673 (93.4%)	75,869 (100.0%)	26,747 (93.6%)	2,057 (37.0%)	0 (0.0%)
High	7,343 (6.6%)	0 (0.0%)	1,827 (6.4%)	3,501 (63.0%)	2,015 (100.0%)
Anxiety symptoms (GAD-2) in 2021					
Low	100,612 (89.8%)	75,869 (100.0%)	24,060 (84.2%)	683 (12.3%)	0 (0.0%)
High	11,404 (10.2%)	0 (0.0%)	4,514 (15.8%)	4,875 (87.7%)	2,015 (100.0%)
Depression and anxiety symptoms (PHQ-4) during COVID-19					
Unchanged	53,978 (50.1%)	40,067 (55.3%)	11,287 (40.4%)	1,991 (36.7%)	633 (31.9%)
Increased	41,436 (38.4%)	22,702 (31.3%)	14,468 (51.7%)	3,041 (56.1%)	1,225 (61.7%)
Decreased	12,384 (11.5%)	9,664 (13.3%)	2,205 (7.9%)	387 (7.1%)	128 (6.4%)
Unknown	4,218	3,436	614	139	29

ACS Guideline, American Cancer Society Guideline for Cancer Prevention; BMI, Body Mass Index; MET, Metabolic Equivalent of Task; PHQ-4, Patient Health Questionnaire-4; PHQ-2, Patient Health Questionnaire-2; GAD-2, Generalized Anxiety Disorder-2.

^a^Other race and ethnic category include American Indian, Alaska Native, and a write-in option.

^b^The scoring approach for BMI accounts for weight change over time; two measures of BMI were used to calculate the score: 2006–2013 enrollment and 2015 follow-up.

^c^Diet score is based on intakes of fruit and vegetables, variety of fruit and vegetables, whole grains, red/processed meat, highly processed food or refined grain, and sugar sweetened beverages. Higher scores indicate higher alignment with the ACS Guideline recommendations for diet.

Across all models, higher ACS Guideline Scores in 2015 were associated with fewer symptoms of depression and/or anxiety in 2021. Participants with higher ACS Guideline Scores had lower odds of experiencing depression and anxiety symptoms (score 7–8: OR: 0.60; 95% CI: 0.57–0.63) compared to those with the lowest scores (0–2; model 1 in Table 2; Supplementary eFigure 1). Excluding participants with pre-existing depression and/or anxiety and individuals taking medications for depression and/or anxiety in 2015 and 2021 attenuated the strength of associations (model 2 in Table 2; Supplementary eFigure 2). Specifically, individuals with higher ACS Guideline Scores experienced lower odds of anxiety and depression symptoms (0.78, 0.73–0.85) compared to lower scoring participants.

**Table 2:** Associations between ACS Guideline Scores and symptoms for depression and anxiety.

ACS Guideline Scores	Model 1			Model 2^b^		
	Adjusted for age, sex, energy intake, and sociodemographic factors^a^			Sensitivity analysis, excluding pre-existing diagnosis and medication use		
	*n*	OR (95% CI)	*p*-value	*n*	OR (95 CI%)	*p*-value
Depression and anxiety symptoms (PHQ-4)						
0–2	14,127	1 [Reference]		6,865	1 [Reference]	
3	15,952	0.89 (0.85–0.94)	<0.001	8,560	0.97 (0.90–1.05)	0.503
4	21,267	0.81 (0.77–0.84)	<0.001	12,338	0.94 (0.87–1.01)	0.086
5	23,671	0.71 (0.68–0.74)	<0.001	14,688	0.87 (0.81–0.94)	<0.001
6	20,227	0.65 (0.62–0.68)	<0.001	12,984	0.84 (0.78–0.90)	<0.001
7–8	16,772	0.60 (0.57–0.63)	<0.001	11,159	0.78 (0.73–0.85)	<0.001
Depressive symptoms (PHQ-2)						
0–2	14,127	1 [Reference]		6,865	1 [Reference]	
3	15,952	0.83 (0.77–0.90)	<0.001	8,560	0.96 (0.79–1.16)	0.654
4	21,267	0.67 (0.62–0.72)	<0.001	12,338	0.80 (0.67–0.96)	0.018
5	23,671	0.55 (0.51–0.60)	<0.001	14,688	0.71 (0.59–0.85)	<0.001
6	20,227	0.48 (0.44–0.52)	<0.001	12,984	0.68 (0.56–0.82)	<0.001
7–8	16,772	0.43 (0.39–0.47)	<0.001	11,159	0.66 (0.54–0.81)	<0.001
Anxiety symptoms (GAD-2)						
0–2	14,127	1 [Reference]		6,865	1 [Reference]	
3	15,952	0.93 (0.87–1.00)	0.044	8,560	1.01 (0.87–1.17)	0.924
4	21,267	0.82 (0.76–0.87)	<0.001	12,338	0.93 (0.81–1.07)	0.290
5	23,671	0.71 (0.67–0.76)	<0.001	14,688	0.87 (0.75–0.99)	0.040
6	20,227	0.67 (0.62–0.72)	<0.001	12,984	0.83 (0.72–0.96)	0.010
7–8	16,772	0.62 (0.57–0.67)	<0.001	11,159	0.84 (0.72–0.97)	0.017

ACS Guideline, American Cancer Society Guideline for Cancer Prevention; PHQ-4, Patient Health Questionnaire-4; PHQ-2, Patient Health Questionnaire-2; GAD-2, Generalized Anxiety Disorder-2; OR, odds ratio.

^a^Sociodemographic factors included race/ethnicity, work status, annual income, education level, and marital status.

^b^Model adjusted for sex, age, energy intake, and all sociodemographic factors.

### Individual behaviors

Individually, all four health behaviors were associated with depression and anxiety symptoms (Table 3). As sub-scores for BMI, PA, and diet increased (reflecting more desirable behavior in each category), symptoms of depression and anxiety decreased. Individuals with an optimal score of 2 for BMI, PA, and diet had lower odds of anxiety and depression symptoms compared to participants with scores of 0 (BMI: 0.70, 0.68–0.73; PA: 0.75, 0.73–0.77; diet: 0.87, 0.84–0.90). When considering depression and anxiety symptoms alone, receiving a score of 2 for BMI (i.e., maintaining a normal BMI) was associated with lower odds of depression (0.54, 0.51–0.57); while PA sub-scores of 2 (i.e., exceeding PA guidelines) were associated with lower odds of anxiety (0.70, 0.67–0.73) compared to individuals scoring 0. Interestingly, individuals with alcohol sub-scores of 1 [i.e., moderate consumption (22)] and 2 (i.e., nondrinkers) had lower odds of depression and anxiety symptoms (0.87, 0.83–0.92 and 0.92, 0.87–0.97, respectively), though individuals with scores of 1 experienced greater protective associations.

**Table 3:** ACS Guideline sub-scores and symptoms for depression and anxiety.

Sub-scores		*n*	Depression and anxiety symptoms (PHQ-4)		Depressive symptoms (PHQ-2)		Anxiety symptoms (GAD-2)	
			OR (95% CI)^a^	*p*-value	OR (95% CI)^a^	*p*-value	OR (95% CI)^a^	*p*-value
Body Mass Index	0	33,138	1 [Reference]		1 [Reference]		1 [Reference]	
	1	37,323	0.80 (0.78–0.83)	<0.001	0.70 (0.66–0.74)	<0.001	0.82 (0.78–0.86)	<0.001
	2	41,555	0.70 (0.68–0.73)	<0.001	0.54 (0.51–0.57)	<0.001	0.75 (0.71–0.78)	<0.001
Physical Activity	0	34,209	1 [Reference]		1 [Reference]		1 [Reference]	
	1	11,268	0.87 (0.83–0.91)	<0.001	0.74 (0.68–0.81)	<0.001	0.86 (0.81–0.92)	<0.001
	2	66,539	0.75 (0.73–0.77)	<0.001	0.60 (0.57–0.63)	<0.001	0.70 (0.67–0.73)	<0.001
Diet	0	34,401	1 [Reference]		1 [Reference]		1 [Reference]	
	1	36,550	0.94 (0.91–0.97)	<0.001	0.82 (0.78–0.87)	<0.001	0.94 (0.89–0.99)	0.012
	2	41,065	0.87 (0.84–0.90)	<0.001	0.73 (0.69–0.78)	<0.001	0.88 (0.83–0.92)	<0.001
Alcohol	0	8,763	1 [Reference]		1 [Reference]		1 [Reference]	
	1	73,724	0.87 (0.83–0.92)	<0.001	0.85 (0.77–0.93)	<0.001	0.86 (0.80–0.93)	<0.001
	2	29,529	0.92 (0.87–0.97)	0.003	1.07 (0.97–1.19)	0.167	0.96 (0.88–1.04)	0.299

ACS Guideline, American Cancer Society Guideline for Cancer Prevention; PHQ-4, Patient Health Questionnaire-4; PHQ-2, Patient Health Questionnaire-2; GAD-2, Generalized Anxiety Disorder-2; OR, odds ratio.

^a^Multivariable models were adjusted by age, sex, energy intake, race/ethnicity, work status, annual income, education level, and marital status.

### Associations due to pandemic-related changes in depression and anxiety

During the COVID-19 pandemic, 38% of participants reported increased depression and anxiety symptoms (*n* = 41,436). Table 4 shows results stratified by changes in PHQ-4 scores during COVID-19 to account for the occurrence of the pandemic within study follow-up years (*n* = 107,798). The protective association of aligning to the ACS Guideline in 2015 remained consistent among participants reporting unchanged or increased PHQ-4 scores (*p* < 0.001). Participants whose symptoms decreased during the pandemic had a less clear dose–response (Supplementary eFigure 3) and a weakened association between higher ACS Guideline Scores and symptoms of depression and anxiety (0.77, 0.65–0.92). Furthermore, participants scoring 7–8 with no or increased change in symptoms during the pandemic experienced greater protective effects by aligning to the ACS Guideline compared to their lower scoring counterparts (unchanged: 0.54, 0.50–0.58; increased: 0.61, 0.57–0.66).

**Table 4:** ACS Guideline Scores and PHQ-4 scores, stratified by changes during the COVID-19 pandemic^a^.

ACS Guideline Scores	Symptoms during the pandemic: unchanged (*n* = 53,978)			Symptoms during the pandemic: increased (*n* = 41,436)			Symptoms during the pandemic: decreased (*n* = 12,384)		
	*N*	OR (95% CI)	*p*-value	*N*	OR (95% CI)	*p*-value	*N*	OR (95% CI)	*p*-value
0–2	6,786	1 [Reference]		5,174	1 [Reference]		1,681	1 [Reference]	
3	7,689	0.86 (0.80, 0.92)	<0.001	5,836	0.88 (0.81, 0.95)	<0.001	1,892	1.03 (0.88, 1.21)	0.675
4	10,466	0.78 (0.73, 0.84)	<0.001	7,490	0.80 (0.75, 0.86)	<0.001	2,481	0.92 (0.79, 1.07)	0.274
5	11,481	0.68 (0.64, 0.73)	<0.001	8,663	0.69 (0.64, 0.74)	<0.001	2,589	0.90 (0.77, 1.04)	0.155
6	9,577	0.58 (0.54, 0.63)	<0.001	7,697	0.66 (0.62, 0.71)	<0.001	2,153	0.76 (0.65, 0.89)	<0.001
7–8	7,979	0.54 (0.50, 0.58)	<0.001	6,576	0.61 (0.57, 0.66)	<0.001	1,588	0.77 (0.65, 0.92)	0.004

ACS Guideline, American Cancer Society Guideline for Cancer Prevention; PHQ-4, Patient Health Questionnaire-4; OR, odds ratio.

^a^Multivariable models were adjusted by age, sex, energy intake, race/ethnicity, work status, annual income, education level, and marital status.

## Discussion

This study is the first to our knowledge to examine prospective associations between co-occurring behaviors for BMI, PA, diet, and alcohol consumption recommended by the 2020 ACS Guideline for Cancer Prevention (25) and mental health outcomes. Higher alignment to the 2020 ACS Guideline demonstrated consistent inverse associations with lower odds of future depression and/or anxiety symptoms across all models. These findings suggest that individuals who follow healthier lifestyle behaviors, as captured by higher ACS Guideline Scores, may experience better mental health outcomes over time. Engaging in healthier individual behaviors including maintaining a normal BMI, meeting and exceeding PA guidelines, higher diet quality, and moderate alcohol consumption was consistently associated with lower odds of future symptoms of depression and anxiety. To address the potential of reverse causality and temporality, we demonstrated that while less pronounced, the association between ACS Guideline Scores and PHQ-4 scores were consistent when excluding participants with pre-existing depression and/or anxiety and those taking medication for depression and/or anxiety. The results of this sensitivity analysis were interpreted in the context of our primary findings and reinforced the robustness of our results.

Numerous studies have explored the impact of health behaviors on mental health, though a majority focus on PA (12, 13) and/or are in relation to depression (16), so it is not clear the extent co-occurring health behaviors have on mental health (39). Findings from a meta-analysis found engaging in at least three multiple healthy behaviors, including PA, diet, smoking, BMI, and alcohol consumption, lowered one’s risk of depression by approximately 50% (40). Conversely, a cross-sectional study among women in college found multiple unhealthy behaviors (i.e., poor diet quality, sleep duration, alcohol consumption, and tobacco/nicotine use) were associated with more severe depression and anxiety symptoms (41). Many studies examining co-occurring health behaviors do not specify exact behaviors such as MVPA or food group consumption (i.e., fruit and vegetable consumption, whole grains) and have synthesized behaviors under general/broad categories (i.e., total PA or diet) (17, 18). Moreover, many studies exploring the combined effects of multiple health behaviors assess associations with depression or anxiety, rather than both. Our study addressed many of these gaps by examining longitudinal associations of combined and individual health behaviors with symptoms of depression and anxiety using the ACS Guideline Score, which has demonstrated criterion validity capturing health behaviors (26, 28, 31).

The COVID-19 pandemic disrupted normal routines, bringing on rapid and unforeseen changes to daily life. Some evidence demonstrates the pandemic allowed for more time to engage in protective health behaviors such as preparing food (42) and increased PA (43); while others experienced increased depression and anxiety symptoms (44–46), weight gain (47), sedentary behavior (48), alcohol consumption (49), and worsened dietary habits (50). Participants reporting decreased symptoms of depression and anxiety during COVID-19 presented a less clear dose–response than participants with no or increased symptoms of depression and anxiety. Importantly, only 11% of participants (*n* = 12,384) reported their depression and anxiety symptoms decreased during the pandemic, perhaps reducing statistical precision. COVID-related changes in PA and mental health within CPS-3 have been investigated; compared to those who remained physically active, individuals who were or became inactive during the pandemic reported more symptoms of depression (51). It is possible other pandemic-related changes could be driving the reduced protective associations of ACS Guideline alignment among participants reporting decreased PHQ-4 scores during COVID.

In this study, BMI, PA, and diet showed clear associations with future mental health outcomes when examined individually, supporting existing evidence on the benefits of individual health behaviors on mental health (16, 19, 52). Exceeding PA guidelines and maintaining a normal BMI, both represented by scores of 2, were the most impactful factors associated with reduced symptoms of anxiety and depression, respectively. The pattern of alcohol use was more nuanced: both moderate drinkers (scores of 1) and nondrinkers (scores of 2) showed reduced odds of both symptoms of anxiety and depression (i.e., total PHQ-4 scores), though moderate intake was associated with the strongest protective associations. While moderate alcohol consumption (22) could increase social interaction and protect against depression, studies on moderate alcohol consumption have not proven a direct cause-and-effect link, thus drinking for health benefits is not recommended and clinical trials are necessary to better understand the impacts of moderate alcohol consumption on health (53). The gradient observed in this study suggests that while abstaining from alcohol was not detrimental to mental health, low level consumption may coincide with social or behavioral contexts that support psychological wellbeing as suggested previously (54). Evidence has demonstrated a J-shaped curve between alcohol consumption and health (55), with protective associations decreasing with excessive alcohol consumption (e.g., binge and heavy drinking) but potentially increasing with low/moderate doses (53). Associations between total ACS Guideline Scores and symptoms of anxiety and depression were greater than sub-score associations, further highlighting the synergistic effects of engaging in multiple healthy behaviors.

### Strengths and limitations

Notable strengths of this study include its prospective design incorporating data collected both before and after the start of the pandemic; large sample size; the capacity to control for confounding variables; and the utilization of survey measures that have proven valid and reliable across diverse populations (28, 56, 57). Previous evidence has demonstrated that CPS-3 is geographically diverse (28, 29); however, the predominantly White and educationally advantaged composition of this cohort may limit generalizability to populations experiencing greater diversity in structural and social determinants of mental health that may shape both health behaviors and symptoms of anxiety and depression. Reverse causation bias may exist, but results from a sensitivity analysis excluding participants with pre-existing anxiety and/or depression diagnoses and participants taking medication for depression and/or anxiety across both timepoints remained significant, though attenuated from the main results, suggesting reverse causation may be playing a role in the primary analysis but likely does not fully explain the main findings. Missing data and attrition resulted in the exclusion of a sizeable proportion of participants, primarily due to nonresponse to the 2021 survey and incomplete dietary data necessary to derive the ACS Guideline Score. Attrition may have been differential, with participants experiencing poorer health behaviors or symptoms of anxiety and depression potentially less likely to remain in the analytical sample. Demonstrated in Supplementary eTable 2, there were many similarities in sociodemographic characteristics, but some small differences with the ACS Guideline Score components, PHQ-2, and GAD-2 scores among included vs. excluded participants. Such selection could attenuate observed associations, leading to conservative estimates of associations between guideline alignment and PHQ-4 scores. These sources of bias should be considered when interpreting results. The occurrence of the COVID-19 pandemic over this study’s follow-up years is an additional limitation. To address this, a stratified analysis was conducted to assess differences by self-reported changes in PHQ-4 during COVID-19. However, participants were past the height of the pandemic and retrospectively responded to these questions. Due to the lack of variability across PHQ-4 scores, particularly among moderate and severe symptoms, examining the longitudinal associations of engaging in health behaviors on varying severities of depression and anxiety were unable to be explored. PHQ-4 is not a clinical diagnosis but rather was developed as a quick measure to detect symptoms of depression and anxiety (34). Further research is warranted to explore associations of co-occurring health behaviors and mental health outcomes utilizing diagnostic data.

## Conclusion

The temporal associations identified in this study suggest that adopting multiple healthy behaviors may contribute protective benefits against future poor mental health outcomes. As the prevalence of depression and anxiety continue to increase, investigating the complex relationship between mental health and health behaviors (both independent and co-occurring) remains a public health priority.

## Data Availability

Data are available from the American Cancer Society by following the ACS Data Access Procedures (https://www.cancer.org/research/population-science/research-collaboration.html) for researchers who meet the criteria for access to confidential data. Please email cohort.data@cancer.org to inquire about access. KC and ER-P had full access to all the data in the study and takes responsibility for the integrity of the data and the accuracy of the data analysis.
