# Supplementary material for: Co-occurring health behaviors and mental health outcomes among a large, aging US population
Source: Front Public Health. 2026 May 12;14:1817690. doi: 10.3389/fpubh.2026.1817690 (PMC13201199; doi:10.3389/fpubh.2026.1817690)
Supplement: Supplementary file 1 [file Table_1.docx]

# SUPPLEMENT MATERIAL

## eTable 1. Scoring the Patient Health Questionnaire-4 for Depression and Anxiety (PHQ-4)

| How often have you been bothered by the following problems? | | Not at all | Several Days | More than half the days | Nearly every day |
| --- | --- | --- | --- | --- | --- |
| GAD-2.2 | Feeling nervous, anxious, or on edge | 0 | 1 | 2 | 3 |
| GAD-2.2 | Not being able to stop or control worrying | 0 | 1 | 2 | 3 |
| PHQ-2.1 | Feeling down, depressed, or hopes | 0 | 1 | 2 | 3 |
| PHQ-2.2 | Little interest or pleasure in doing things | 0 | 1 | 2 | 3 |


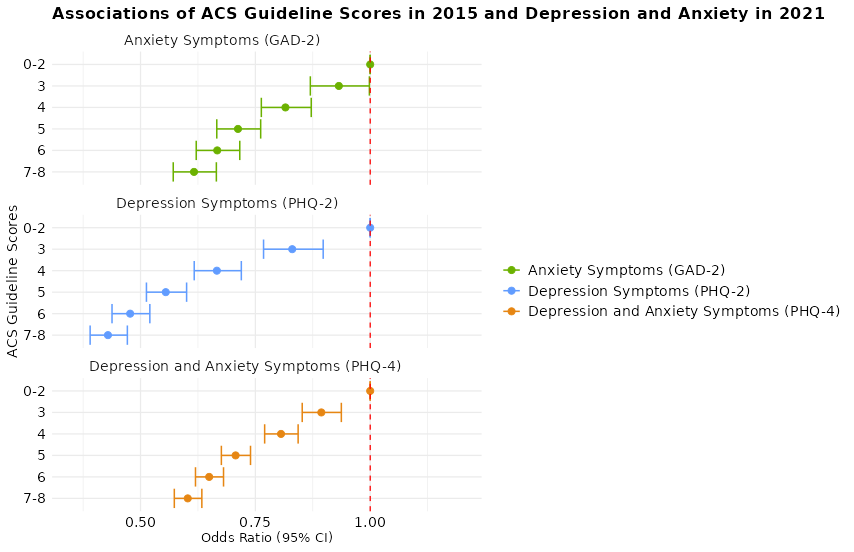


## eFigure 1. Associations of ACS Guideline Scores in 2015 and symptoms of depression and/or anxiety in 2021. Model adjusted for sex, age, energy intake, race/ethnicity, annual income, work status, education level, and marital status.


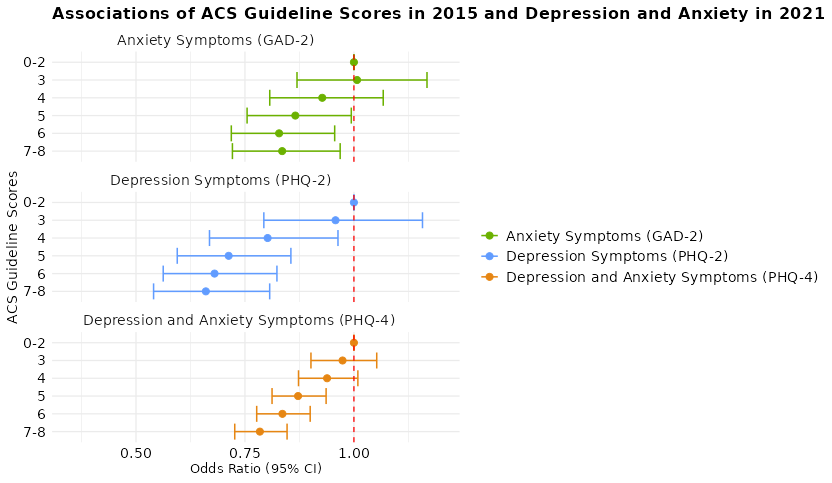


## eFigure 2. Associations ACS Guideline Scores and future depression and/or anxiety excluding participants with pre-existing diagnosis for depression and/or anxiety and those taking medication for depression and/or anxiety in 2015 and 2021. Model adjusted for sex, age, energy intake, race/ethnicity, annual income, work status, education level, and marital status.


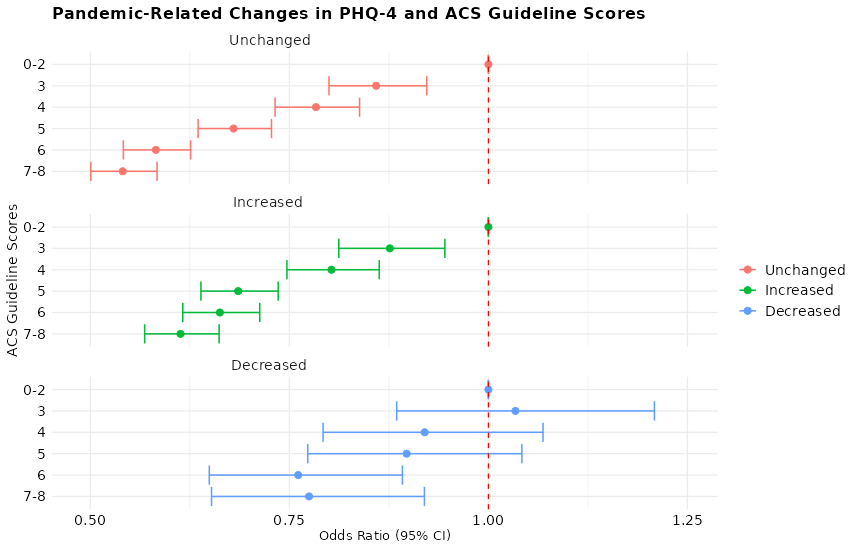


## eFigure 3. Associations of ACS Guideline Scores in 2015 and PHQ-4 in 2021 stratified by pandemic-related changes in depression and anxiety. Model adjusted for sex, age, energy intake, race/ethnicity, annual income, work status, education level, and marital status.
