# Supplementary material for: Co-occurring health behaviors and mental health outcomes among a large, aging US population
Source: Front Public Health. 2026 May 12;14:1817690. doi: 10.3389/fpubh.2026.1817690 (PMC13201199; doi:10.3389/fpubh.2026.1817690)
Supplement: Supplementary file 2 [file Table_2.docx]

**Supplement Material**

# eTable 2. Baseline Characteristics between Excluded vs Included Participants

| **Characteristic** | **Excluded  (n = 74,622)** | **Included  (n = 112,016)** |
| --- | --- | --- |
| **Age, mean (SD)** | 52 (10) | 53 (9.5) |
| Unknown | 3 |  |
| **Sex** |  |  |
| Female | 59,482 (79.7%) | 88,643 (79.1%) |
| Male | 15,140 (20.3%) | 23,373 (20.9%) |
| **Race and ethnicity** |  |  |
| Asian, Native Hawaiian, Pacific Islander, or American Indian | 2,326 (3.2%) | 2,741 (2.4%) |
| Black | 2,988 (4.1%) | 2,207 (2.0%) |
| Latino | 5,456 (7.5%) | 5,753 (5.1%) |
| White | 61,488 (84.6%) | 100,863 (90.0%) |
| Other^a^ | 423 (0.6%) | 452 (0.4%) |
| Unknown | 1,941 |  |
| **Work status** |  |  |
| Full time | 48,829 (65.4%) | 75,344 (67.3%) |
| Part time | 8,477 (11.4%) | 13,626 (12.2%) |
| Retired | 8,824 (11.8%) | 13,919 (12.4%) |
| Other | 6,802 (9.1%) | 7,047 (6.3%) |
| Unknown/missing | 1,690 (2.3%) | 2,080 (1.9%) |
| **Income, $** |  |  |
| <50,000 | 15,536 (20.8%) | 15,709 (14.0%) |
| 50,000 to <75,000 | 13,795 (18.5%) | 19,312 (17.2%) |
| 75,000 to <100,000 | 12,589 (16.9%) | 19,732 (17.6%) |
| 100,000 to <125,000 | 10,856 (14.5%) | 18,294 (16.3%) |
| 125,000 or more | 20,148 (27.0%) | 37,269 (33.3%) |
| Unknown/missing | 1,698 (2.3%) | 1,700 (1.5%) |
| **Education level** |  |  |
| High school or less | 7,651 (10.3%) | 6,945 (6.2%) |
| Some college or 2-y degree | 23,527 (31.5%) | 27,906 (24.9%) |
| Graduate degree | 19,228 (25.8%) | 39,908 (35.6%) |
| College graduate | 23,616 (31.6%) | 36,961 (33.0%) |
| Unknown/missing | 600 (0.8%) | 296 (0.3%) |
| **Marital status** |  |  |
| Married or living with partner | 55,619 (74.5%) | 86,093 (76.9%) |
| Never married | 5,592 (7.5%) | 8,364 (7.5%) |
| Divorced, separated, or widowed | 12,565 (16.8%) | 16,258 (14.5%) |
| Unknown/missing | 846 (1.1%) | 1,301 (1.2%) |
| **ACS Guideline Scores (count)** |  |  |
| 0-2 | 9,230 (16.5%) | 14,127 (12.6%) |
| 3 | 9,096 (16.2%) | 15,952 (14.2%) |
| 4 | 11,285 (20.2%) | 21,267 (19.0%) |
| 5 | 11,268 (20.1%) | 23,671 (21.1%) |
| 6 | 8,803 (15.7%) | 20,227 (18.1%) |
| 7-8 | 6,302 (11.3%) | 16,772 (15.0%) |
| Unknown | 18,638 |  |
| **BMI score^b^** |  |  |
| 0 (≥30.0 kg/m^2^ at any time point) | 26,061 (37.3%) | 33,138 (29.6%) |
| 1 (Other combinations) | 23,082 (33.0%) | 37,323 (33.3%) |
| 2 (18.5 - <25.0 kg/m^2^ at both time points) | 20,742 (29.7%) | 41,555 (37.1%) |
| Unknown | 4,737 |  |
| **Physical activity score** |  |  |
| 0 (<7.5 MET-h/wk) | 25,206 (34.9%) | 34,209 (30.5%) |
| 1 (7.5 - <15.0 MET-h/wk) | 7,062 (9.8%) | 11,268 (10.1%) |
| 2 (≥15 MET-h/wk) | 39,945 (55.3%) | 66,539 (59.4%) |
| Unknown | 2,409 |  |
| **Diet score^c^** |  |  |
| 0 (first tertile) | 22,294 (35.0%) | 34,401 (30.7%) |
| 1 (second tertile) | 21,109 (33.1%) | 36,550 (32.6%) |
| 2 (third tertile) | 20,313 (31.9%) | 41,065 (36.7%) |
| Unknown | 10,906 |  |
| **Alcohol score** |  |  |
| 0 (>1 drink/d for women, >2 drinks/d for men) | 5,475 (7.7%) | 8,763 (7.8%) |
| 1 (≤1 drink/d for women, ≤2 drinks/d for men) | 43,613 (61.1%) | 73,724 (65.8%) |
| 2 (0 drink/d) | 22,335 (31.3%) | 29,529 (26.4%) |
| Unknown | 3,199 |  |
| **Depression symptoms (PHQ-2) in 2021** |  |  |
| Low | 28,074 (91.8%) | 104,673 (93.4%) |
| High | 2,522 (8.2%) | 7,343 (6.6%) |
| Unknown | 44,026 |  |
| **Anxiety symptoms (GAD-2) in 2021** |  |  |
| Low | 27,028 (87.9%) | 100,612 (89.8%) |
| High | 3,712 (12.1%) | 11,404 (10.2%) |
| Unknown | 43,882 |  |
| **Depression and Anxiety (PHQ-4) during COVID-19** |  |  |
| Unchanged | 14,343 (19.2%) | 53,978 (50.1%) |
| Increased | 11,232 (15.1%) | 41,436 (38.4%) |
| Decreased | 4,241 (5.7%) | 12,384 (11.5%) |
| Unknown/missing | 44,806 (60.0%) | 4,218 |

Abbreviations: ACS Guideline, American Cancer Society Guideline for Cancer Prevention; BMI, Body Mass Index; MET, Metabolic Equivalent of Task; PHQ-4, Patient Health Questionnaire-4; PHQ-2, Patient Health Questionnaire-2; GAD-2, Generalized Anxiety Disorder-2.

^a^ Other race and ethnic category include American Indian, Alaska Native, and a write-in option.

^b^ The scoring approach for BMI accounts for weight change over time; two measures of BMI were used to calculate the score: 2006-2013 enrollment and 2015 follow-up.

^c^ Diet score is based on intakes of fruit and vegetables, variety of fruit and vegetables, whole grains, red/processed meat, highly processed food or refined grain, and sugar sweetened beverages. Higher scores indicate higher alignment with the ACS Guideline recommendations for diet.
